# Influence of Cu^2+^ on Osteoclast Formation and Activity In Vitro

**DOI:** 10.3390/ijms22052451

**Published:** 2021-02-28

**Authors:** Anne Bernhardt, Jana Bacova, Uwe Gbureck, Michael Gelinsky

**Affiliations:** 1 Centre for Translational Bone Joint and Soft Tissue Research, TU Dresden, Medical Faculty and University Hospital “Carl Gustav Carus”, 01307 Dresden, Germany; Jana.Bacova@upce.cz (J.B.); Michael.Gelinsky@tu-dresden.de (M.G.); 2 Department for Functional Materials in Medicine and Dentistry, University of Würzburg, 97070 Würzburg, Germany; uwe.gbureck@fmz.uni-wuerzburg.de

**Keywords:** osteoclast, in vitro, TRAP, cathepsin K, resorption, dentin, copper

## Abstract

Background: Copper-containing biomaterials are increasingly applied for bone regeneration due to their pro-angiogenetic, pro-osteogenetic and antimicrobial properties. Therefore, the effect of Cu^2+^ on osteoclasts, which play a major role in bone remodeling was studied in detail. Methods: Human primary osteoclasts, differentiated from human monocytes were differentiated or cultivated in the presence of Cu^2+^. Osteoclast formation and activity were analyzed by measurement of osteoclast-specific enzyme activities, gene expression analysis and resorption assays. Furthermore, the glutathione levels of the cells were checked to evaluate oxidative stress induced by Cu^2+^. Results: Up to 8 µM Cu^2+^ did not induce cytotoxic effects. Activity of tartrate-resistant acid phosphatase (TRAP) was significantly increased, while other osteoclast specific enzyme activities were not affected. However, gene expression of TRAP was not upregulated. Resorptive activity of osteoclasts towards dentin was not changed in the presence of 8 µM Cu^2+^ but decreased in the presence of extracellular bone matrix. When Cu^2+^ was added to mature osteoclasts TRAP activity was not increased and resorption decreased only moderately. The glutathione level of both differentiating and mature osteoclasts was significantly decreased in the presence of Cu^2+^. Conclusions: Differentiating and mature osteoclasts react differently to Cu^2+^. High TRAP activities are not necessarily related to high resorption.

## 1. Introduction

The trace element copper (Cu^2+^) is involved in many physiological processes due to its ability to change oxidation states. It has been shown to promote angiogenesis [1] and osteogenesis [2] and shows antimicrobial effects [3]. Cu^2+^ is involved in bone formation and mineralization, as it serves as cofactor of several enzymes like lysyl oxidase, which is responsible for the cross-linking of collagen fibrils [4,5]. Therefore, copper has been widely used in the development of bone graft materials like mesoporous bioactive glasses [6,7], calcium phosphate cements and ceramics [8,9,10,11], titanium implants [12,13,14], chitosan [15,16], collagen [17] and alginate [18]. Potential bone graft materials are commonly tested for their in vitro biocompatibility and for their ability to support growth and osteogenic differentiation of osteoblasts or osteoprogenitor cells. However, there is growing interest to evaluate new bone graft materials with respect to their influence on osteoclast formation and/or osteoclastic resorption in vitro to get first information on remodeling of the materials. In a previous study we investigated Cu^2+^ doped brushite forming calcium phosphate bone cements (together with cements doped with other bioactive metal ions) with respect to their influence on osteoclastic differentiation and resorption [19]. It was shown, that Cu^2+^ had cytotoxic effects on osteoclasts at a concentration of 17.7 µM. In contrast, human mesenchymal stroma cells tolerated Cu^2+^ concentrations up to 250 µM without any retardation of proliferation [11]. Therefore, Cu^2+^ containing materials might be useful to shift the equilibrium between osteoblastic bone formation and osteoclastic resorption to increase the amount of new bone formed around a copper-releasing implant material. The high concentration of copper released by the cements in the previous study prevented the examination of its effects on osteoclastogenesis and functionality, since the cells did not survive the treatment. Investigations on the effect of Cu^2+^ on osteoclastogenesis and resorption are nevertheless of interest to evaluate the effect of biomaterials releasing lower amounts of this ion. The present study aimed to analyze the effect of Cu^2+^ on the activity of osteoclast-specific enzymes tartrate resistant acid phosphatase (TRAP) and cathepsin K as well as the influence on osteoclastic resorption. Some of our preliminary investigations concerning this matter revealed a highly significant increase of TRAP activity, when osteoclasts were cultivated in the presence of Cu^2+^. These unexpected results encouraged us to further study the effect of Cu^2+^ on osteoclast functionality.

TRAP is a very specific osteoclast marker enzyme and its serum level can be correlated to bone resorption [20]. It was shown in a previous study, that the activity of TRAP and other typical osteoclastic enzymes like cathepsin K (CTSK) and carbonic anhydrase II (CA II) can be correlated to some extent to the resorptive activity of osteoclasts in vitro [21]. Therefore, we hypothesized, that the very high TRAP activity of osteoclasts in the presence of low Cu^2+^ concentrations may lead to higher resorption. To test this hypothesis, we differentiated osteoclasts from human peripheral blood mononuclear cells (PBMC) on tissue culture polystyrene (TCPS) as well as dentin slices and on cell-derived extracellular bone matrix in the presence of low Cu^2+^ concentrations. The influence of Cu^2+^ on the osteoclastic enzyme activity, expression of the corresponding osteoclastic enzyme genes and on resorption was studied.

## 2. Results

### 2.1. Cytotoxicity of Cu^2+^ towards PBMC and Osteoclasts

**Figure 1:**
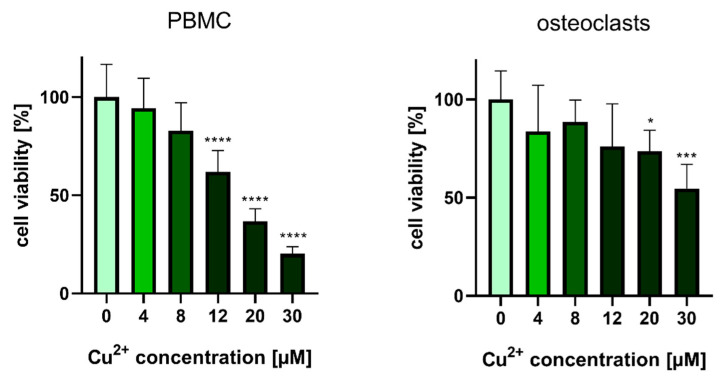
Cell viability of human PBMC and mature human osteoclasts after treatment with Cu^2+^. Luminescence-based CellTiterGlo^®^ assay was used to determine the ATP content, which correlates with the number of metabolically active cells. Cells of two donors with each *n* = 3 (*n* = 6 in total per group) were included into the experiments. Statistic differences were calculated by one-way ANOVA, followed by Tukey’s test for multiple comparisons * *p* < 0.05, *** *p* < 0.001, ****p < 0.0001.

Osteoclasts are more sensitive to Cu^2+^ than mesenchymal stroma cells, which tolerate Cu^2+^ concentrations up to 250 µM without any decrease in cell number [11]. To ensure, that the investigations on osteoclast formation and functionality will be performed in a non-cytotoxic range, viability studies with PBMC and mature osteoclasts were conducted (Figure 1) PBMC showed significant loss of viability at Cu^2+^ concentration of 12 µM and above. In the presence of 30 µM Cu^2+^ the number of viable cells was reduced to 20.3 +/− 3.6%. Interestingly, mature osteoclasts tolerated Cu^2+^ much better. Only at Cu^2+^ concentrations of 20 µM and above a significantly reduced number of viable cells were detected and with 30 µM the number of viable cells was 43.9 +/−9.3 % (Figure 1). The following investigations were performed with Cu^2+^ in concentrations between 0 and 8 µM.

### 2.2. Effect of Cu^2+^ on Osteoclast Number and Activity of Osteoclast-Specific Enzymes

**Figure 2:**
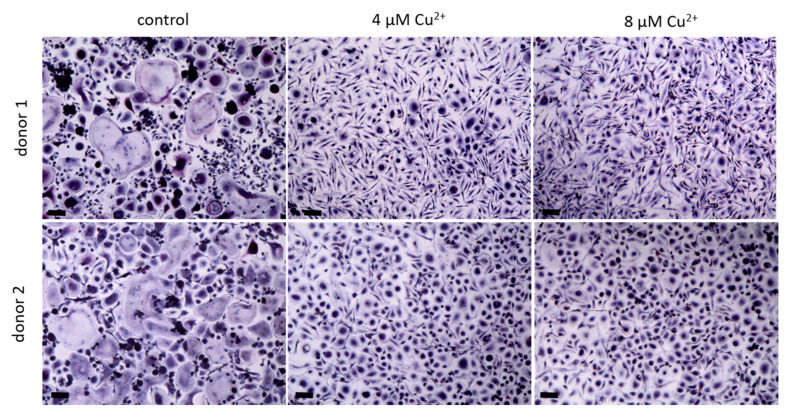
Osteoclasts were differentiated from PBMC for 14 days in the presence of MCSF and RANKL and without Cu^2+^ (control) as well as under the addition of 4 and 8 µM Cu^2+^, light microscopy after staining of TRAP (pink) and nuclei (blue), scale bars represent 100 µm.

**Figure 3:**
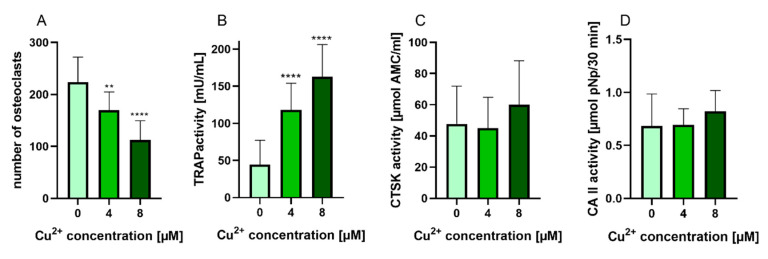
(**A**) Number of multinucleated TRAP positive osteoclasts differentiated from human PBMC after 14 days of cultivation, counted from light microscopic images (cells of two donors, xx images per condition) Statistic differences were calculated by one-way ANOVA, followed by Tukey’s test for multiple comparisons (**B**) TRAP activity, (**C**) CTSK activity and (**D**) CAII activity of osteoclasts differentiated from human PBMC after 14 days of cultivation. Data of 7 different donors (each *n* = 5) were included into the calculation. (*n* = 35 in total). Statistical differences were calculated using Kruskal Wallis Test **** *p* < 0.0001.

PBMC were differentiated under stimulation with M-CSF and RANKL to osteoclasts in the presence of 4 and 8 µM Cu^2+^. Multinucleated TRAP positive cells were formed in all groups (Figure 2) however with increasing copper concentration the number of osteoclasts decreased significantly (Figure 3A). As already found in our preliminary investigations, activity of TRAP significantly increased in the presence of Cu^2+^ (Figure 3B), while the activities of cathepsin K and CAII activity remained on the same level (Figure 3C,D).

### 2.3. Effects of Cu^2+^ on Gene Expression of Osteoclast Markers

**Figure 4:**
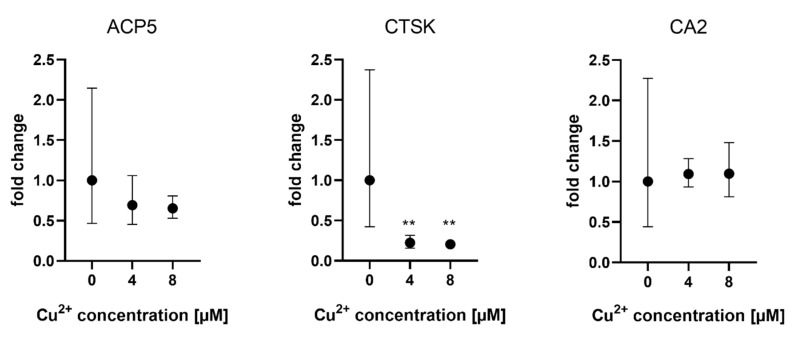
Expression of osteoclast marker genes in PBMC derived osteoclasts after 14 days of differentiation. Fold changes were calculated by the ΔΔCT method and related to the samples without Cu^2+^. Samples of three different PBMC donors were used (each *n* = 3, *n* = 9 in total per group), mean ± upper/lower limit. Statistical differences were calculated from DCT values. ** *p* < 0.01.

Gene expression of osteoclast markers ACP5 (TRAP), CTSK and CAII was analyzed when osteoclasts were differentiated in the presence of MSCF/RANKL and Cu^2+^. While TRAP and CA2 expression were not affected by Cu^2+^, CTSK expression was slightly reduced in the presence of Cu^2+^ (Figure 4).

Control values without addition of Cu^2+^ displayed high variances between the cells of the three different donors (see Supplemental Figure S1).

### 2.4. Effects of Cu^2+^ on Resorption

**Figure 5:**
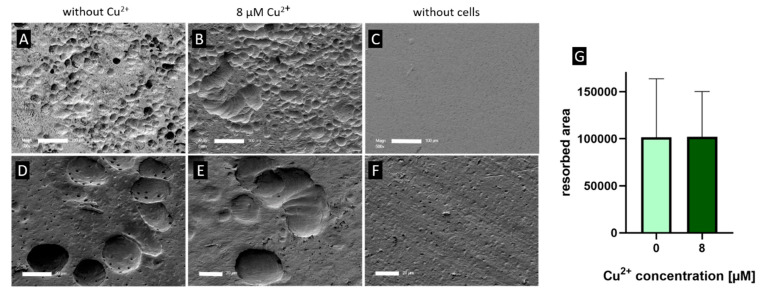
Representative SEM images of resorption pits after 14 days cultivation of PBMC derived osteoclasts on dentine slices (**A**,**D**) control, (**B**,**E**) 8 µM Cu^2+^, (**F**,**G**) without cells, scale bar represent 100 µm (**A**–**C**) and 20 µm (**D**–**F**). (**G**) Open source Fiji software was used to calculate resorbed area of dentin slices (cells of two PBMC donors, 24 images per group). For details see Supplemantary Figure S2. Mann-Whitney did not reveal significant differences between 0 and 8 µM Cu^2+^.

Resorption of dentin slices was not changed when osteoclasts were cultivated in the presence of Cu^2+^ (Figure 5). Nevertheless, TRAP activity of the osteoclasts on dentin slices was again significantly higher in the presence of Cu^2+^ (data not shown).

**Figure 6:**
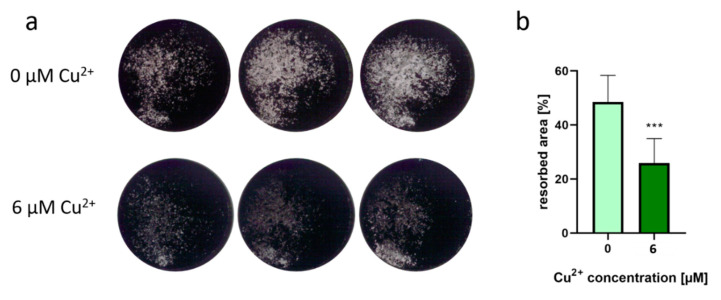
(**a**) Representative images of osteoblast-derived ECM after osteoclastic resorption in the presence of 6 µM Cu^2+^ compared to a copper-free control. SaOS-2 osteoblasts were cultivated for 4 weeks until a closed layer of mineralized extracellular matrix was formed in the dishes. After removal of the osteoblasts, PBMC (two donors, each *n* = 4 per group) were seeded and cultivated for 14 days under stimulation with M-CSF and RANKL with and without addition of Cu^2+^. After fixing with 4% formaldehyde, von Kossa staining was performed to stain the remaining mineralized matrix after osteoclastic resorption. Images were recorded with a Leica stereomicroscope and represent the whole area of a 48- well dish (12 mm diameter). (**b**) Resorbed area of all samples was calculated applying the open source software Fiji using the threshold function. Eight samples were imaged for each condition and shown as mean +/− standard deviation. Significant differences were calculated by two-tailed unpaired *t*-test with *p* < 0.001.

In contrast, resorption of mineralized extracellular bone matrix was significantly reduced when osteoclasts were differentiated in the presence of Cu^2+^ (Figure 6).

### 2.5. Effect of Cu^2+^ on Mature Osteoclasts

**Figure 7:**
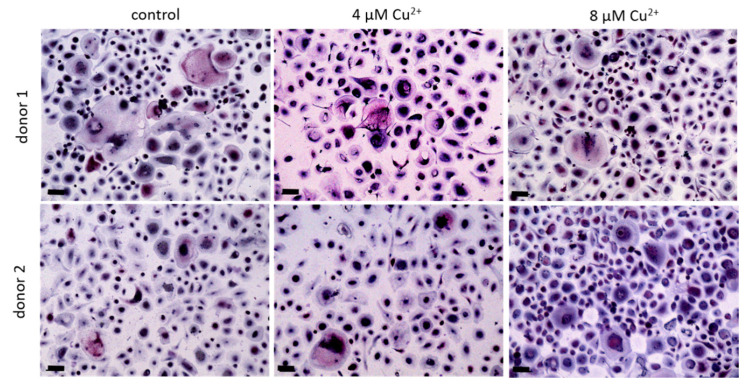
Osteoclasts were differentiated from PBMC for 10 days in the presence of MCSF and RANKL, detached and cultivated for further 4 days without Cu^2+^ (control) as well as under the addition of 4 and 8 µM Cu^2+^, light microscopy after staining of TRAP (pink) and nuclei (blue), scale bars represent 100 µm.

**Figure 8:**
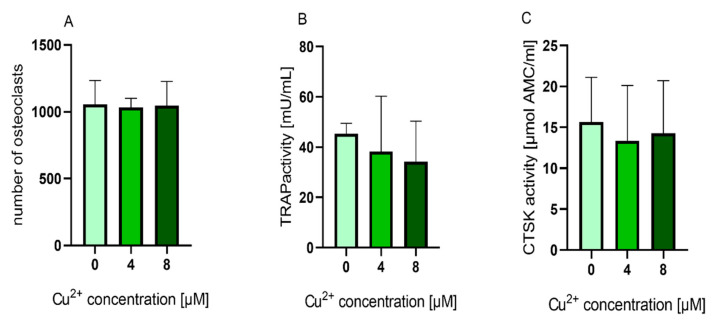
(**A**) Number of multinucleated TRAP positive osteoclasts counted from light microscopic images (cells of two donors, 4 images per condition) Statistic differences were calculated by one-way ANOVA, followed by Tukey’s test for multiple comparisons (**B**) TRAP activity and (**C**) CTSK activity of mature osteoclasts cultivated in the presence of 0, 4 and 8 µM Cu^2+^. Data of four different donors were included into the calculation with each *n* = 3 replicates per group (average +/− standard deviation). TRAP and CTSK activities were normalized to the copper free control, since the enzyme activity levels between the different donors showed high variances. Statistical significant differences were calculated by one way ANOVA followed by Tukey’s test to perform multiple comparisons. * *p* < 0.05 compared to the control.

We were interested to find out, whether the TRAP activity increasing effect of Cu^2+^ was also detectable, when the ion was applied to cultures of already differentiated osteoclasts. Mature osteoclasts (after 10–11 days of differentiation) were detached from the surfaces, were the PBMC were originally seeded and further cultivated in the presence of MCSF, RANKL and Cu^2+^. Interestingly, the number of TRAP positive osteoclasts did not decrease in the presence of Cu^2+^ (Figure 7 and Figure 8A) Furthermore, no significant increase of TRAP activity was detected. (Figure 8).

**Figure 9:**
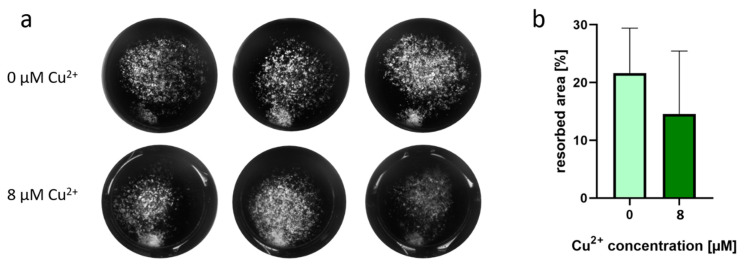
(**a**) Representative images of osteoblast-derived ECM after osteoclastic resorption in the presence of 8 µM Cu^2+^ compared to a copper-free control. SaOS-2 osteoblasts were cultivated for 4 weeks until a closed layer of mineralized extracellular matrix was formed in the dishes. After removal of the osteoblasts, mature osteoclasts, detached from suspension dishes (two donors, each *n* = 3 per group) were seeded and cultivated for 4 days under further stimulation with M-CSF and RANKL with and without addition of 8 µM Cu^2+^. After fixing with 4% formaldehyde, von Kossa staining was performed to stain the remaining mineralized matrix after osteoclastic resorption. Images were recorded with a Leica stereomicroscope and represent the whole area of a 48- well dish (12 mm diameter). (**b**) Resorbed area of all samples was calculated applying the open source software Fiji using the threshold function. Six samples were imaged for each condition and are shown as mean +/− standard deviation. Two-tailed unpaired t-test did not show significant differences between 0 and 8 µM Cu^2+^.

Resorption of osteoblast mineral matrix by mature osteoblasts was not changed in the presence of Cu^2+^ (Figure 9).

### 2.6. Effect of Cu^2+^ on Glutathione Levels

**Figure 10:**
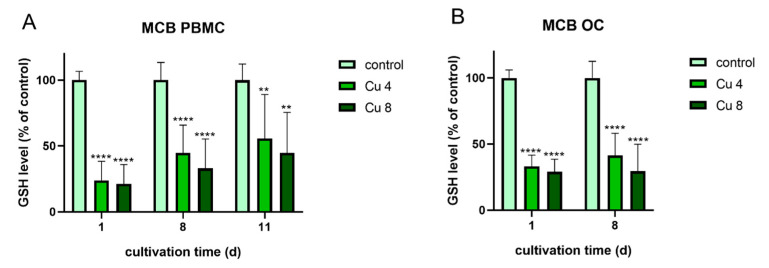
Glutathione levels of PBMC during osteoclastic differentiation (**A**) and of mature osteoclasts (**B**) in the presence of 4 and 8 µM Cu^2+^ compared to a copper-free control. Data of three different PBMC donors (each *n* = 3; *n* = 9 in total per group and time point) were included into the calculations. Statistically significant differences were calculated by 2-way ANOVA followed by Tukey’s multiple comparisons test * *p* < 0.05, ** *p* < 0.01, *** *p* < 0.001, **** *p* < 0.0001.

The following experiments were performed to find out, whether oxidative stress, which was induced due to the presence of Cu^2+^ might be in correlation to the increased TRAP activity. For this reason both differentiating PBMC and already differentiated mature osteoclasts were treated again with 4 and 8 µM Cu^2+^ and the glutathione levels of the cells were determined using the monochlorbimane (MCB) assay (Figure 10).

Glutathione levels of both osteoclasts and PBMC were significantly reduced in the presence of Cu^2+^. The highest reduction was detected for PBMC after one day of cultivation. This reduction cannot be attributed to the reduction in osteoclast number, since it was decreased only in differentiating osteoclasts (Figure 3A) and stayed stable in mature osteoclasts in the presence of Cu^2+^ (Figure 8A).

Interestingly, mature osteoclasts, which did not increase their TRAP activity in the presence of Cu^2+^, nevertheless showed a significant reduction of glutathione levels.

## 3. Discussion

The present study analyses the effect of Cu^2+^ on the activity of osteoclast-specific enzymes, their gene expression and on osteoclastic resorption. Cytotoxicity studies revealed a higher sensitivity of PBMC compared to mature osteoclasts towards Cu^2+^. Löffler and co-workers investigated the effect of metal ions on monocytes and macrophages. They postulated that macrophages might be better equipped against the destructive effects of metal ions, since they keep closer contact to metal ions during phagocytosis. Accordingly, Löffler et al. could show significantly higher metabolic activities of macrophages compared to monocytes at low Co^2+^ concentrations (Cu^2+^ was not included into this study) [22]. Similar effects might be conceivable for monocytes and osteoclasts in the presence of the metal ion Cu^2+^.

We demonstrated a significantly increased TRAP activity, when PBMC were differentiated into osteoclasts in the presence of 4 and 8 µM Cu^2+^ and this effect was consistently found for PBMC of more than 15 different donors. In contrast, gene expression of TRAP (ACP5) as well as osteoclastic resorption was not increased in the presence of Cu^2+^; resorption was even slightly inhibited by the ion. To rule out an influence of Cu^2+^ on the fluorimetric TRAP assay, we tested the assay with cell lysates, which were obtained from PBCM derived osteoclasts and added 10 µM Cu^2+^ directly to the assay. However, the TRAP activity was not increased in the presence of Cu^2+^, it was even decreased to around 70% compared to samples without copper (data not shown). Furthermore, our results show that TRAP gene expression was not increased, when PBMC were differentiated into osteoclasts in the presence of 4 and 8 µM Cu^2+^. There are not many investigations on the influence of Cu^2+^ on osteoclastogenesis and osteoclastic resorption in the literature. As early as 1981 Wilson and co-workers cultivated mouse calvaria tissue in the presence and absence of Cu^2+^ and showed a dose-dependent decrease of osteoclastic resorption [23]. In 2002 Zhang et al. demonstrated an inhibition of resorption, when rabbit osteoclasts were cultivated on bone slices in the presence of 1 µM Cu^2+^ [24]. Finally, the group of Pamela Habibovic deposited different elements, including copper to calcium phosphate films in a biomimetic approach. They demonstrated a significantly reduced resorption when primary rabbit osteoclasts were cultivated on copper-containing films [25]. All these studies did not analyse TRAP activity, however, a reduced resorption in the presence of Cu^2+^ was also demonstrated in our study, but only in the case of osteoclasts, which were treated with Cu^2+^ during osteoclastogenesis from PBMC and only towards extracellular bone matrix (Figure 6). Besides differentiating osteoclasts, which were formed from PBMC, also mature osteoclasts were involved. In a previous study [26] we compared different surfaces for the detachment of osteoclasts, which generally resulted in relatively low yields of detached osteoclasts. Heinemann and co-workers also report difficulties in detaching osteoclasts, which were formed in vitro on commonly used cell culture surfaces and propose special temperature-sensitive dishes for osteoclast detachment [27]. In the present study, we tried ultra-low attachment cell culture flasks for formation and detachment of osteoclasts. Both differentiation time and osteoclast yield were considerably increased on these surfaces compared to other cell culture surfaces. PMBC were able to attach loosely to the surfaces, which might have promoted their ability to migrate and fuse. After being formed, the multinucleated osteoclasts could be easily detached just by adding low concentrations of buffered EDTA without using a cell scraper. Interestingly, in our study the resorption was less affected, when Cu^2+^ was added to mature osteoclasts, indicating an effect of Cu^2+^ on osteoclast formation rather than osteoclast function. However, the above cited study of Yang et al. also used mature osteoclasts, directly isolated from bone and found a significantly decreased osteoclastic resorption.

The central questions of this study are how Cu^2+^ affects TRAP activity and why both TRAP gene expression and osteoclastic resorption are not increased in the same way. TRAP activity is regulated in a redox dependent pathway: the active centre contains two ferric ions, and the enzyme is active, when one of them is in a reduced state [28]. Copper as redox active metal ion is able to generate reactive oxygen species, which could change the redox state of TRAP and therefore activate this enzyme. This would be in line with the unchanged TRAP mRNA expression in the presence of Cu^2+^, indicating a regulation on protein level. However, it has been shown that copper is a potent inhibitor of TRAP activity with a Ki of 6.8 µM [29] which is in line with our observation of a reduced TRAP activity when copper ions are added directly to the assay (see above). It has been shown in numerous studies that copper can induce oxidative stress in biological systems [30,31]. Intracellular antioxidant substances protect cells from highly reactive oxygen species (ROS) generated by different redox reactions. The most prevalent cellular protective antioxidant is the thiol-containing tripeptide glutathione, which is present in all cells in high concentration [32]. Different methods were proposed to quantify the level of reduced glutathione (GSH) in cells. Among these, the fluorometric monochlorbimane assay [33], which was optimized by Capek, and co-workers is a useful method since the reagent is able to penetrate through the cell membrane and to directly react with intracellular GSH [34]. We demonstrated a significant reduction of GSH levels in both PBMC and mature osteoclasts, indicating, that the cells suffered from oxidative stress in the presence of low concentrations of Cu^2+^. It has been early postulated, that increased ROS levels support osteoclastogenesis and TRAP activity [35,36]. Later, different groups reported an elevation of intracellular ROS level during stimulation of monocytes with RANKL and reduced osteoclast formation when the cultures were treated with antioxidants [37,38]. ROS generated through the presence of Cu^2+^ could therefore have increased TRAP activity in our experiments. However, why did the increased TRAP activity not imply an increased osteoclastic resorption? It has been shown before, that TRAP activity does not necessarily correlate with osteoclastic resorption. The function of TRAP in osteoclasts is proposed to further degrade collagen fragments, which were initially digested by cathepsin K, and it was shown, that the enzyme itself is able of producing ROS for these degradation processes [39]. It has been shown, that TRAP activity is rather a marker for osteoclast number than for osteoclast resorptive activity [40,41]. In addition, our own investigations displayed a strong dependency of TRAP activity from the composition of the resorbed material [21]. In the case of Cu^2+^ or Cu^2+^ containing biomaterials it can be concluded that the highly significant increase of TRAP activity is not reflected by an increased osteoclastic activity. Quite the opposite, a reduction of resorptive activity of osteoclasts in vitro was observed in the presence of Cu^2+^ for osteoclast, differentiated from PBMC in the presence of extracellular bone matrix (Figure 6). The reduced number of osteoclasts in this experimental setup (Figure 2 and Figure 3A) can explain this effect. In contrast, the addition of Cu^2+^ to already differentiated osteoclasts did decrease neither osteoclast number nor resorptive activity (Figure 8A and Figure 9).

Consequently, Cu^2+^ released from potential implant materials in concentrations below 10 µM does not induce increased osteoclastic resorption. As shown in our study, higher concentrations of Cu^2+^ are cytotoxic to osteoclasts and PBMC in any case, while mesenchymal stromal cells were shown to tolerate Cu^2+^ concentrations up to 250 µM [11]. Therefore, doping of bone graft materials with low amounts of Cu^2+^ could shift the equilibrium between bone formation and bone resorption around the implant.

In a conclusion, Cu^2+^ has a substantial influence on the formation of osteoclasts from PBMC. While considerably less multinucleated osteoclasts are formed in the presence of even low amounts of Cu^2+^ (4 and 8 µM), the activity of the most specific osteoclastic enzyme, TRAP, is significantly increased. In this study, we could show that this increased TRAP activity is neither based on an increased gene expression nor contributed to an increased osteoclastic resorption. We hypothesized, that ROS, generated by Cu^2+^ induced the detected high TRAP activity. In contrast to PBMC mature osteoclasts are less sensitive to Cu^2+^. Higher Cu^2+^ concentrations were necessary to reduce cell viability of mature osteoclasts and low Cu^2+^ concentrations did not trigger the activity of TRAP.

## 4. Materials and Methods

### 4.1. Osteoclast Cell Culture

#### 4.1.1. Isolation of PBMC and Osteoclast Formation

*Buffy coats* were purchased from the German Red Cross Dresden and PBMC were isolated by density gradient centrifugation, followed by lysis of erythrocytes as previously described [42]. Monocyte content of the PBMC was analyzed using an automated cell counter (scepter 2.0., Merck Millipore, Darmstadt, Germany). Cells containing 5 × 10^5^ monocytes were seeded into 48 well plates and supplemented with α-MEM containing 10% heat inactivated fetal calf serum (Corning, a trademark of Thermo Fisher Scientific, Waltham, MA, USA), 2 µM L-glutamine and 100 U/mL penicillin and 100 µg/mL streptomycin (all from Gibco, a trademark of Thermo Fisher Scientific, Waltham, MA, USA) (PS) (adhesion medium). After one day of cultivation the medium was changed to α-MEM, 5% heat inactivated FCS, 5% human serum, L-Glu, PS, 25 ng/mL MCSF and 50 ng/mL RANKL (both from Peprotech, Hamburg, Germany) (osteoclast differentiation medium). Cu^2+^ containing medium was prepared by adding different volumes of a 3 mM stock solution of Cu(NO_3_)_2_ (Sigma-Aldrich, Munich, Germany) in water to the respective medium. For the generation of osteoclasts PBMC were cultivated for 14 days with medium changes twice per week.

#### 4.1.2. Generation and Transfer of Mature Osteoclasts

PBMC containing 10^7^ monocytes were seeded to ultra-low attachment cell culture flasks 25 cm^2^ (Corning, a trademark of Thermo Fisher Scientific) in adhesion medium, medium was changed to osteoclast differentiation medium the next day and the cells were further cultivated for 10–11 days. Monocytes and differentiated osteoclasts adhered to the flasks while all other cells were washed away during media changes. After formation of multinucleated cells the medium was aspirated, cell layer was washed with PBS and the flasks were shaken with 2 mM EDTA and 0.5 % BSA in PBS for 20 min at room temperature to detach the osteoclasts. Cell suspension was centrifuged, the pellet was washed with PBS and cells were seeded to fresh well plates and again supplemented with osteoclast differentiation medium (with and without addition of Cu^2+^).

### 4.2. Analysis of Cu^2+^ Concentration in the Medium

The final Cu^2+^ concentration of the media was checked by ICP OES (Plasma Quant Elite, Analytik Jena, Jena, Germany). To this end, the different media were diluted with 2% nitric acid in deionized water and the copper content was analyzed with respect to a calibration line composed from a commercially available standard solution (ICP multielement standard IV, Merck-Millipore). The copper content of medium without addition of Cu^2+^ (1.14 µM) was subtracted from all Cu^2+^ containing media, since this amount of copper comes from the FCS and is mostly bound to serum proteins [43].

### 4.3. Cytotoxicity Assay

PBMC and mature osteoclasts were seeded into white 96 well plates using adhesion medium. After 24 h, the medium was changed to differentiation medium containing different amounts of Cu^2+^ (0–30 µM). After another 24 h, cell viability was determined using the luminescent CellTiterGlo^®^ assay (Promega, Walldorf, Germany) according to manufacturer’s instructions.

### 4.4. TRAP Staining

0.3 mg/mL Fast Red Violet LB and 0.1 mg/mL naphthol AS-MX phosphate disodium salt (both Sigma-Aldrich, Munich, Germany) were dissolved in 100 mM sodium acetate buffer pH 6.1 containing 50 mM disodium tartrate (Sigma-Aldrich, Munich, Germany). Cells were fixed with 4% formaldehyde and incubated for 10 min at 37 °C with the staining solution. After washing with PBS, cell nuclei were stained for 10 min using Mayer’s Haemalum solution (AppliChem, Darmstadt, Germany) followed by rinsing in tap water. Stained samples were imaged using a Biorevo BZ-9000 (Keyence, Neu-Isenburg, Germany). For counting of multinucleated TRAP positive cells, three wells (48 well size) per culture condition and PBMC donor were analysed with each 4 images (20× magnification) for each sample. The cells were counted manually using the cell counter plugin of Fiji.

### 4.5. Quantification of TRAP, CTSK and CA2 Activity

After 14 days of cultivation (osteoclasts from PBMC) or 8 days of cultivation (mature osteoclasts detached from flasks), the samples were washed with PBS and frozen at −80 °C. After thawing, lysis buffer (1% Triton × 100 in PBS) was added and the samples were incubated on ice for 50 min. During this incubation step 10 min of ultrasonic treatment in ice water were performed. Activity of osteoclastic enzymes was analysed from the lysates as previously described [21].

For TRAP activity 10 μL of lysate were incubated with 50 μL of 2.5 mM naphthol-ASBI-phosphate disodium salt hydrate (Sigma-Aldrich, Munich, Germany) in 100 mM sodium acetate and 50 mM disodium tartrate pH 6.1 and incubated at 37 °C for 30 min. After stopping the enzymatic reaction (150 µL 0.1 M NaOH) the intensity of fluorescence was measured at an excitation and emission wavelength of 405/520 nm. Calibration solutions, with different TRAP concentrations (0.6 to 12 U/L) taken from a commercially available ELISA kit (BoneTRAP, Medac, Wedel, Germany), were used to correlate the fluorescence intensity with TRAP activity.

For quantification of CAII activity 50 µL of cell lysates were mixed with 50 µL 2 mM 4-nitrophenylacetate in 12.5 mM TRIS pH 7.5 and 75 mM NaCl. Absorbance was monitored at 400 nm for 5 min. Conversion to 4-nitrophenol was calculated from the slope of the absorbance plot using a calibration line from different dilutions of 1 mM 4-nitrophenol (Sigma-Aldrich, Munich, Germany).

For CTSK activity, a 10 mM stock solution of Z-LR-AMC (Enzo Life Sciences, Lörrach, Germany) in DMSO was dissolved 1:100 in 0.1 M sodium acetate buffer containing 4 mM EDTA and 4 mM DTT at pH 5.5. 10 μL of cell lysate plus 40 µL lysis buffer were mixed with 50 µL of this substrate solution and incubated for 30 min at 37 °C. Fluorescence was measured at an excitation/emission wavelength of 365/440 nm. The amount of released aminomethylcoumarin was quantified with a calibration line.

### 4.6. Generation of Extracellular Matrix and Resorption Studies

Osteoblast-derived native extracellular matrix was prepared as published by Lutter et al. [44]. SaOS-2 osteoblasts (DSMZ, Braunschweig, Germany) were seeded in 48-well TCPS (1 × 10^4^ cells/well). After one week of cultivation in α-MEM containing 10% FCS, PS and L-Glu the medium was further supplemented with 100 nM dexamethasone, 5 mM β-glycerophosphate and 12.5 μg/mL ascorbic acid-2 phosphate (all from Sigma-Aldrich, Munich, Germany). Cells were cultivated for 4 weeks until a closed layer of mineralised extracellular matrix was formed in the dishes. Cells were removed by addition of 15 mM ammonium hydroxide solution and washed three times with PBS. PBMC or mature osteoclasts were seeded and supplemented with osteoclast differentiation medium with and without Cu^2+^. After 14 (PBMC) and 8 days of cultivation, samples were fixed with 4% formaldehyde. Staining of fixed samples was performed with 5% AgNO_3_ in deionized water for 30 min under exposure to light. Samples were imaged with a Leica stereomicroscope. Resorbed area was calculated applying the open source software Fiji using the threshold function. Eight samples were imaged for each condition.

### 4.7. Resorption of Dentin

Dentin slices were prepared from canine teeth of minipigs. Teeth were sawed into discs of 0.8 mm thickness using an electric diamond saw and sterilized by gamma irradiation. PBMC of two different donors containing 3 × 10^5^ monocytes/cm^2^ were seeded to the discs and cultivated with the same protocol as described in Section 4.1. in the presence and absence of 8 µM Cu^2+^. After 14 days of cultivation, cells were removed by freezing/thawing and ultrasonication in the presence of 1% Triton X100 in PBS. Samples were dehydrated with increasing amounts of ethanol, dried in a heat cabinet at 37 °C for several days and finally sputtered with gold. SEM images were taken using a Philips XL 30/ESEM with FEG (field emission gun), operated in scanning electron microscopy (SEM) mode: Eight independent SEM images per sample with 500× magnification were used for the calculation of resorption area applying the open source software Fiji (for details see Supplemental Figure S2).

### 4.8. Gene Expression Analysis

RNA was isolated from osteoclasts after 14 days of differentiation using a commercially available kit (peqGOLD MicroSpin Total RNA Kit; Peqlab, Erlangen, Germany). cDNA was generated using the High-Capacity cDNA Reverse Transcription Kit (Applied Biosystems, trademark of Thermo Fisher Scientific) according to manufacturer’s instructions. Polymerase chain reaction (PCR) reactions were set up using the TaqMan Fast Advanced Master Mix (Applied Biosystems) and TaqMan Gene Expression Assays (Applied biosystems) for glyceraldehyde-3-phosphate dehydrogenase (GAPDH) as housekeeping gene as well as, tartrate-resistant acid phosphatase (ACP5), cathepsin K (CTSK) and carbonic anhydrase II (CA2) according to manufacturer’s instructions. PCR was run with an Applied Biosystems 7500 fast real-time PCR system. Relative gene expression (ΔCT) was normalized to GAPDH. Fold changes of gene expression for copper-treated samples were calculated using the ΔΔCT method. Osteoclasts of three PBMC donors were included (*n* = 3, 6 cDNA samples per group). Statistical differences were calculated from ΔCT values by one-way ANOVA followed by Tukey’s multiple comparisons test. ** *p* < 0.01.

### 4.9. Measurement of GSH Levels

GSH levels in cultures of PBMC and osteoclasts were measured using the optimized monochlorbimane (MCB) assay as previously described [34]. For these experiments, PBMC and mature osteoclasts were seeded into black 96 well plates and cultivated with 100 µL osteoclast differentiation medium with and without addition of Cu^2+^. Working solution of monochlorbimane (Sigma-Aldrich, Munich, Germany) was diluted with PBS and tempered at 37 °C. After cultivation, 20 µL of this solution was added to the cells directly before measurement yielding a final MCB concentration of 40 µM in a well. Changes of the fluorescence signal were recorded at Ex/Em 394/490 nm over a period of 20 min.

## Data Availability

All data generated or analysed during this study are included in this published article and its Supplemental Data.

## Supplementary Materials

Supplementary Materials can be found at https://www.mdpi.com/1422-0067/22/5/2451/s1. Figure S1: relative expression of osteoclast markers in osteoclasts of three PBMC donors. Controls withgout addition of Cu^2+^. Figure S2: Resorbed area of dentin slices was calculated applying the open source software Fiji. The “oval selections” tool was used to manually mark all visible resorp-tion pits (yellow circles). After marking all pits, the marked area was measured.

## Abbreviations

M-CSF	Macrophage colony-stimulating factor
RANKL	Receptor activator of nuclear factor kappa-Β ligand
PBS	Phosphate buffered saline
BSA	Bovine serum albumin
PBMC	Peripheral blood mononuclear cells
TRAP	Tartrate resistant acid phosphatase
CTSK	Cathepsin K
GSH	Glutathione
