# Supplementary material for: Influence of Cu2+ on Osteoclast Formation and Activity In Vitro
Source: Int J Mol Sci. 2021 Feb 28;22(5):2451. doi: 10.3390/ijms22052451 (PMC7957576; doi:10.3390/ijms22052451)
Supplement: Supplementary file 1 [file ijms-22-02451-s001.pdf]

Supplemental material:

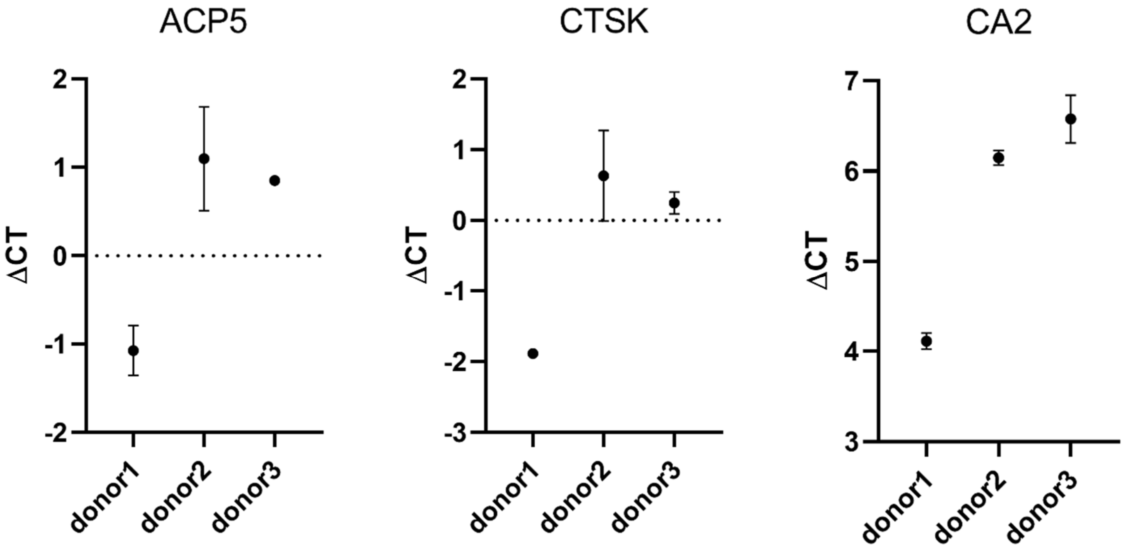

Figure S1: relative expression of osteoclast markers in osteoclasts of three PBMC donors. Controls withgout addition of Cu2+.

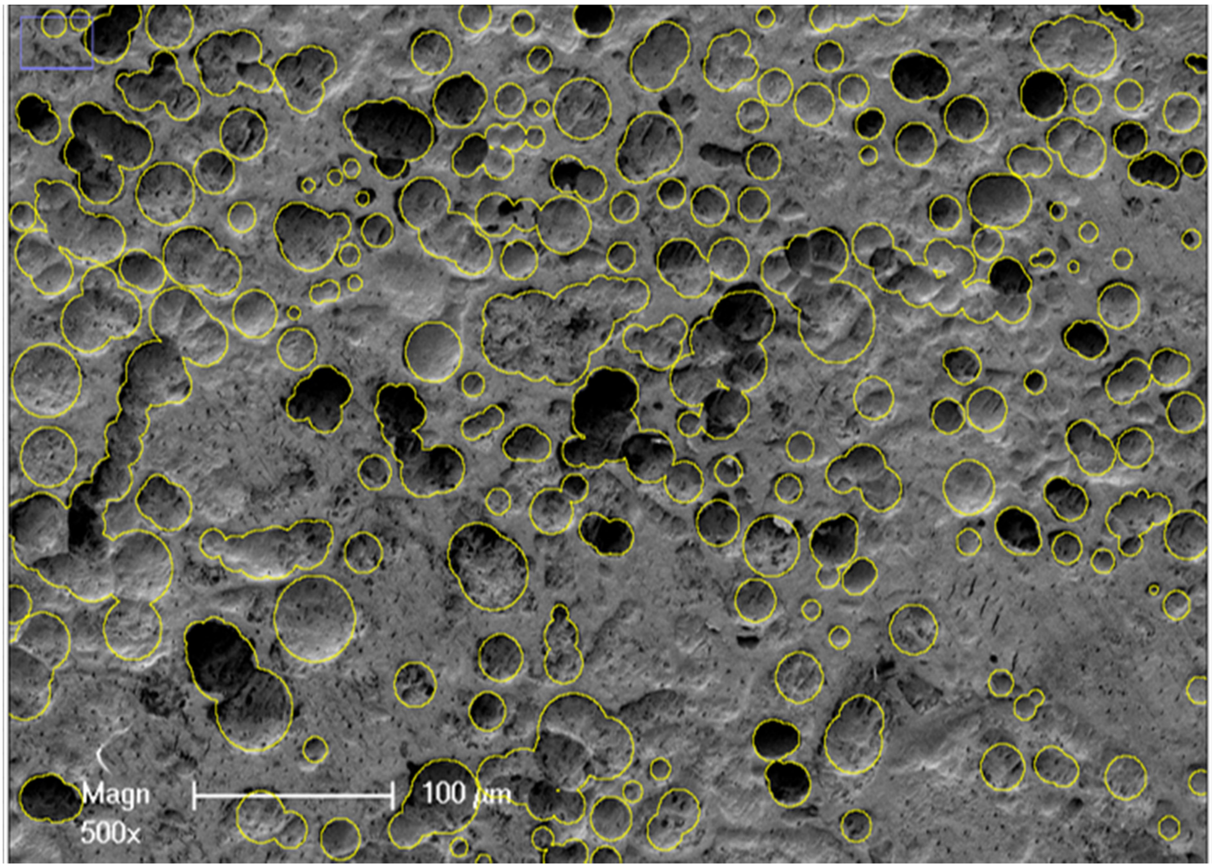

Figure S2: Resorbed area of dentin slices was calculated applying the open source software Fiji. The „oval selections“ tool was used to manually mark all visible resorption pits (yellow circles). After marking all pits, the marked area was measured.

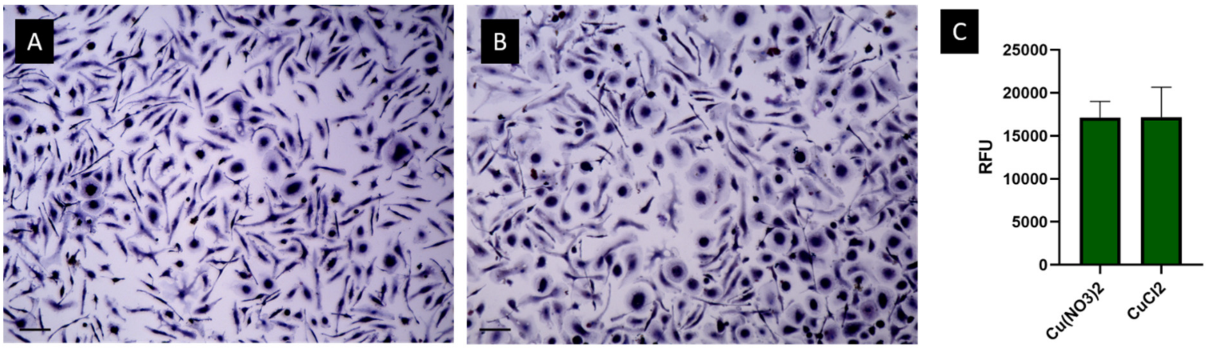

Figure S3: Osteoclasts were differentiated from PBMC for 14 days in the presence of MCSF and RANKL and w under the addition of 8 µM $Cu(NO_3)_2$ (A) or 8 µM $CuCl_2$ (B) , light microscopy after staining of TRAP (pink) and nuclei (blue), scale bars represent 100 µm C TRAP activity of osteoclasts after 14 days of differentiation in the presence of $Cu(NO_3)_2$ and $CuCl_2$ (two PBMC donors, each n=3).
